# SARS-CoV-2 Infected Pediatric Cerebral Cortical Neurons: Transcriptomic Analysis and Potential Role of Toll-like Receptors in Pathogenesis

**DOI:** 10.3390/ijms22158059

**Published:** 2021-07-28

**Authors:** Agnese Gugliandolo, Luigi Chiricosta, Valeria Calcaterra, Mara Biasin, Gioia Cappelletti, Stephana Carelli, Gianvincenzo Zuccotti, Maria Antonietta Avanzini, Placido Bramanti, Gloria Pelizzo, Emanuela Mazzon

**Affiliations:** 1 IRCCS Centro Neurolesi “Bonino-Pulejo”, Via Provinciale Palermo, Contrada Casazza, 98124 Messina, Italy; agnese.gugliandolo@irccsme.it (A.G.); luigi.chiricosta@irccsme.it (L.C.); placido.bramanti@irccsme.it (P.B.); 2 Department of Pediatrics, Ospedale dei Bambini “Vittore Buzzi”, 20154 Milano, Italy; valeria.calcaterra@unipv.it (V.C.); gianvincenzo.zuccotti@unimi.it (G.Z.); 3 Pediatrics and Adolescentology Unit, Department of Internal Medicine, University of Pavia, 27100 Pavia, Italy; 4 Department of Biomedical and Clinical Sciences–L. Sacco, University of Milan, 20157 Milan, Italy; mara.biasin@unimi.it (M.B.); gioia.cappelletti@unimi.it (G.C.); gloriapelizzo@gmail.com (G.P.); 5 Pediatric Clinical Research Center Fondazione Romeo ed Enrica Invernizzi, University of Milan, 20157 Milan, Italy; stephana.carelli@unimi.it; 6 Cell Factory, Pediatric Hematology Oncology Unit, Fondazione IRCCS Policlinico San Matteo, 27100 Pavia, Italy; ma.avanzini@smatteo.pv.it; 7 Pediatric Surgery Unit, Ospedale dei Bambini “Vittore Buzzi”, 20154 Milano, Italy

**Keywords:** SARS-CoV-2, nervous system, children, transcriptomic analysis, human neuronal cells, Toll-like receptors

## Abstract

Different mechanisms were proposed as responsible for COVID-19 neurological symptoms but a clear one has not been established yet. In this work we aimed to study SARS-CoV-2 capacity to infect pediatric human cortical neuronal HCN-2 cells, studying the changes in the transcriptomic profile by next generation sequencing. SARS-CoV-2 was able to replicate in HCN-2 cells, that did not express *ACE2*, confirmed also with Western blot, and *TMPRSS2*. Looking for pattern recognition receptor expression, we found the deregulation of scavenger receptors, such as SR-B1, and the downregulation of genes encoding for Nod-like receptors. On the other hand, *TLR1*, *TLR4* and *TLR6* encoding for Toll-like receptors (TLRs) were upregulated. We also found the upregulation of genes encoding for ERK, JNK, NF-κB and Caspase 8 in our transcriptomic analysis. Regarding the expression of known receptors for viral RNA, only RIG-1 showed an increased expression; downstream RIG-1, the genes encoding for TRAF3, IKKε and IRF3 were downregulated. We also found the upregulation of genes encoding for chemokines and accordingly we found an increase in cytokine/chemokine levels in the medium. According to our results, it is possible to speculate that additionally to ACE2 and TMPRSS2, also other receptors may interact with SARS-CoV-2 proteins and mediate its entry or pathogenesis in pediatric cortical neurons infected with SARS-CoV-2. In particular, TLRs signaling could be crucial for the neurological involvement related to SARS-CoV-2 infection.

## 1. Introduction

COVID-19 is a severe acute respiratory disease caused by the coronavirus SARS-CoV-2. Since the first cases of infection registered in Wuhan, China in late 2019, it spread widely all over the world becoming a global pandemic. The clinical manifestations of COVID-19 can vary going from an asymptomatic infection to mild, moderate, and ultimately severe respiratory illness with multi-organ dysfunctions that may lead to the death of the patient [1]. The most common symptoms and signs are fever, cough, dyspnea, fatigue/myalgia, anosmia and difficulty breathing and also lymphopenia may be present [2]. Airways, and in particular, the oral cavity and nostrils, represent the access route for SARS-CoV-2 and protective personal equipment can be used to stop virus spreading also among high risk jobs [3]. 

An increasing number of studies reported abnormalities of the central nervous system (CNS) and the peripheral nervous systems (PNS) in adult patients with COVID-19 [4,5,6,7]. Hyposmia is a common neurological symptom of COVID-19 that in the worst cases culminates in anosmia. Very different neurological manifestations were observed after SARS-CoV-2 infections such as headache, dizziness, impaired consciousness, Guillain–Barré Syndrome [8], ageusia, seizure, encephalitis, visual and oculomotor impairment, facial palsy [9,10,11,12,13,14,15] but also stroke [16]. Additionally, different neuropsychiatric symptoms, including anxiety disorders, mood disorders, psychosis, and insomnia, have been described [17]. It is important to notice that neurological symptoms may continue also after recovery [18]. Indeed, some patients showed anosmia, headache and other symptoms and signs also after recovery [19,20]. 

Different mechanisms were proposed to explain neurological symptoms caused by SARS-CoV-2 but a clear one was not established yet [21]. Possible mechanisms to explain how SARS-CoV-2 can enter the CNS are the hematogenous or neuronal retrograde dissemination. In the hematogenous entry route, a virus can infect endothelial cells of the blood–brain barrier or leukocytes for dissemination into the CNS. The second entry route into the CNS suggested that viruses infect neurons in the periphery and use the axonal transport machinery to enter the CNS [22]. Cranial nerve and olfactory nerve represent another potential route of entry into the CNS for SARS-CoV-2 [13]. Some coronaviruses can invade the CNS by penetrating the anatomical structures of ethmoid affecting the olfactory bulb ability to control viral invasion and supporting a retrograde transsynaptic propagation. Another retrograde route of propagation may be mediated by SARS-CoV-2 invasion of sensorial receptors located in lung and airways [23]. It has also been proposed that SARS-CoV-2 can invade brain via the vagal afferents from the gastrointestinal tract, suggesting a role for gut–brain axis [24].

Interestingly, SARS-CoV-2 was found in the cerebrospinal fluid (CSF) [25] and in the brains of some patients [26]. Other than the neuroinvasive potential of SARS-CoV-2, it is necessary also to clarify if ACE2 represents the main route of entry of SARS-CoV-2 into neuronal cells. Indeed, neuronal ACE2 expression is still unclear [27,28]. However, it is possible to speculate that also other receptors can be used by SARS-CoV-2 to enter the host cells [29].

Evidence suggests that combined direct and indirect mechanisms play a role in developing CNS and PNS involvement. As reported [30], different patho-physiological processes may be implicated such as neurotropic properties of SARS-CoV-2, damage to microvasculature, brainstem compromise, neuroinflammatory response, cytokine storm, autoimmune response, demyelination, systemic hypoxia.

Data supporting the SARS-CoV-2 PNS and CNS neurological involvement are limited in pediatric age. Children account for only 1–5% of COVID-19 cases, of which more than 80% are asymptomatic or mild cases [31,32,33,34]. About 25–50% of children, depending on the studies, with “multisystem inflammatory syndrome in children” (MIS-C) related to SARS-CoV-2 showed neurological manifestations [31,35,36].

In this study, we performed a next generation sequencing analysis in order to evaluate the transcriptional changes induced by SARS-CoV-2 infection in pediatric human cortical neurons (HCN-2). The aim was to elucidate potential pathways involved in SARS-CoV-2 infection of neuronal cells. The discovery of SARS-CoV-2 mechanisms of infection may help to prevent nervous system early and long term complications in children and to define better pediatric treatments and prognosis. 

## 2. Results

### 2.1. SARS-CoV-2 Replication in Neuronal Cells

**Figure 1:**
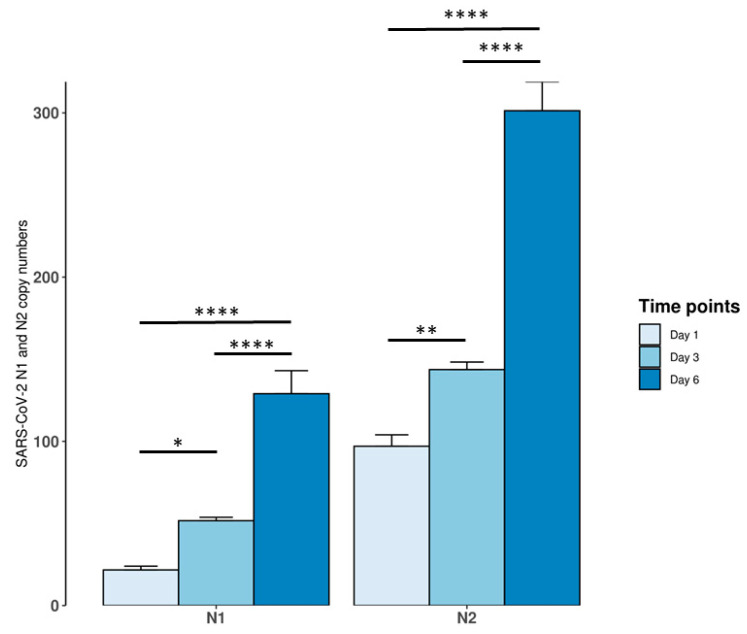
Virus replication in HCN-2 cortical neurons. N1 and N2 copy number increased in a time dependent manner. * *p* < 0.05; ** *p* < 0.01; **** *p* < 0.0001.

In order to verify if SARS-CoV-2 was able to replicate in neuronal HCN-2 cells we evaluated N1 and N2 copy number at 3 different time point, namely 1, 3 and 6 days post infection. We observed a time dependent increase in N1 and N2 copy numbers, indicating that the virus was able to replicate in HCN-2 neuronal cells (Figure 1). 

### 2.2. SARS-CoV-2 Modulated Pathways Related to Viral and Bacterial Infections 

**Figure 2:**
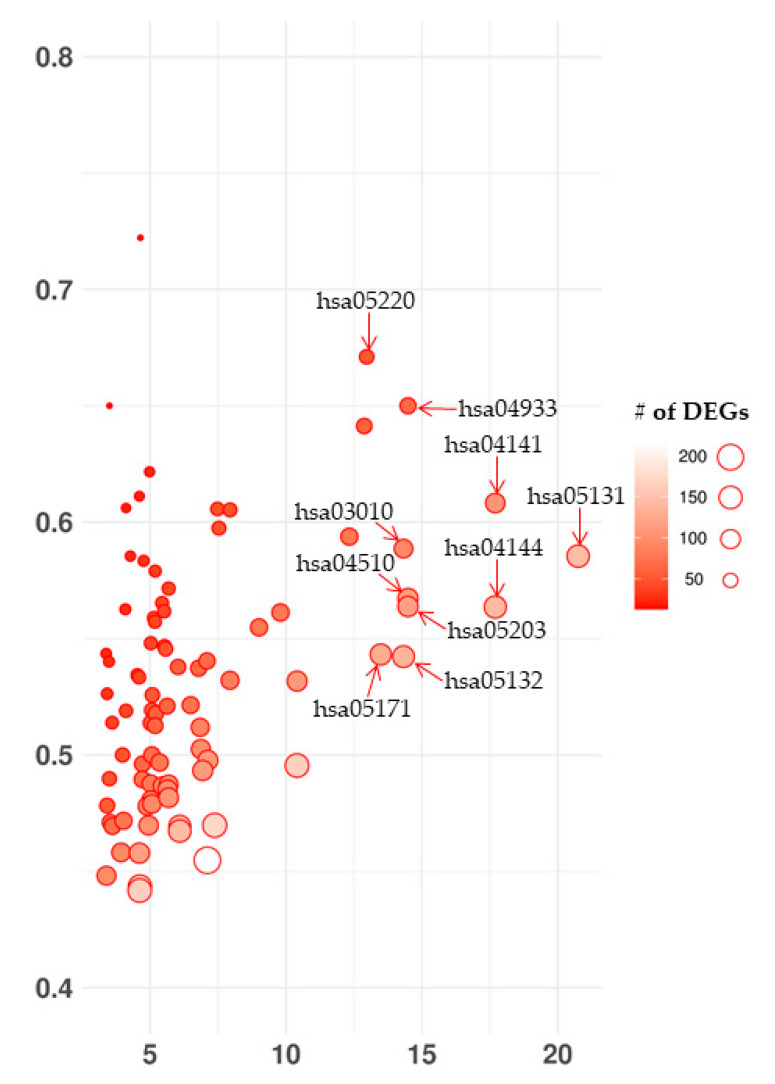
Bubble plot of enriched KEGG maps of DEGs in transcriptomic analysis of HCN2-CTR against HCN2-SARS-CoV-2. The pathways are vertically sorted by the ratio of the number of DEGs observed in the map over the total amount of genes that take place there. Ideally, if all the genes in the map are deregulated, the value is 1, while no DEGs in the map is 0. The size of the circle gives information about the number of DEGs included in the pathway. From the left to the right we represented a score for each pathway obtained by −log(*q*-Value). Interestingly, the “Coronavirus disease—COVID-19” (hsa05171) is among the first 10 overrepresented maps. Then, “Shigellosis” (hsa05131), “Protein processing in endoplasmic reticulum” (hsa04141), “Endocytosis” (hsa04144), “AGE-RAGE signaling pathway in diabetic complications” (hsa04933), “Focal adhesion” (hsa04510), “Viral carcinogenesis” (hsa05203), “Ribosome” (hsa03010), “Salmonella infection” (hsa05132) and “Chronic myeloid leukemia” (hsa05220) have the highest score.

No cut-off was chosen to define the differentially expressed genes (DEGs) but only genes with *q*-values lower than 0.05 were inspected. The transcriptomic analysis of non-infected (HCN2-CTR) against SARS-CoV-2-infected HCN-2 cells (HCN2-SARS-CoV-2) revealed 7315 DEGs. Among them, 3527 were more expressed in HCN2-SARS-CoV-2 while 3788 were more expressed in HCN2-CTR. We enriched our data with KEGG maps and we observed 96 pathways overrepresented that are plotted in the bubble plot in Figure 2. Notably, the “Coronavirus disease—COVID-19” was among the first 10 overrepresented maps. Then, “Shigellosis”, “Protein processing in endoplasmic reticulum”, “Endocytosis”, “AGE-RAGE signaling pathway in diabetic complications”, “Focal adhesion”, “Viral carcinogenesis”, “Ribosome”, “Salmonella infection” and “Chronic myeloid leukemia” had the highest score based on −log(*q*-Value).

### 2.3. SARS-CoV-2 Modulated Pattern Recognition Receptor Expression

**Figure 3:**
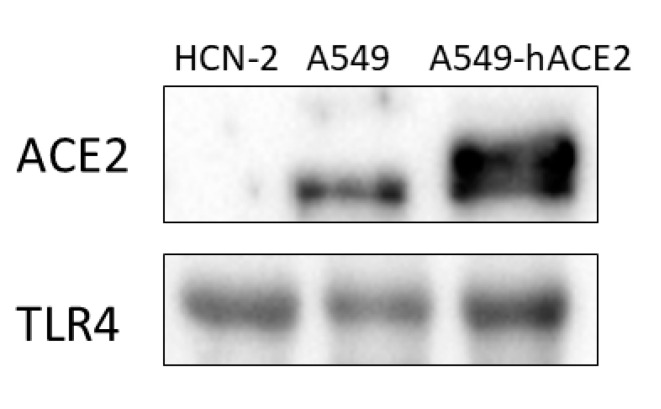
Western blot for ACE2 and TLR4. HCN-2 cells expressed TLR4 but not ACE2. A549 and A549-hACE2 expressed ACE2 and TLR4.

At first, we evaluated if HCN-2 cells expressed the receptors used by SARS-CoV-2 to enter into the cells. *ACE2* and *TMPRSS2* that encode, respectively, for the proteins Angiotensin-converting enzyme 2 and Transmembrane protease, serine 2 were not expressed in our transcriptomic analysis. Their role in COVID-19 is widely documented because ACE2 allows the binding of SARS-CoV-2 in the lung cells [37] and TMPRSS2 acts as protein priming for the virus [38]. For this reason, the Human Protein Atlas and the Gene Expression Atlas were used to confirm that none of them are detected in the human cerebral cortex. Moreover, we performed Western blot analysis in order to verify if HCN-2 cell line expressed ACE2 protein. We also evaluated ACE2 protein in A549 cell line and in A549 cells expressing Human ACE2 (hACE2). We observed that HCN-2 cells did not express ACE2 that was instead expressed in both A549 cells and in A549-hACE2 (Figure 3). 

The cells use the pattern recognition receptors (PRRs) in order to recognize pathogens and start the innate immune response. Table 1 collects the PRRs deregulated in the analysis. The membrane-bound PRRs include 13 genes among which *TLR1*, *TLR4* and *TLR6* are Toll-like receptors (TLRs) while *AGER*, *CD163L1*, *CD36*, *MEGF10*, *SCARB1*, *SCARB2*, *SCARF1*, *SSC5D*, *MRC1* and *OLR1* are Scavenger receptors. On the other hand, 3 cytoplasmic PRRs were deregulated. *DDX58* is a Rig-like receptor (RLRs) while *NLRC5* and *NLRP1* belong to the Nod-like receptors (NLRs) family. In addition, we showed that HCN-2 cells expressed TLR4 by western blot (Figure 3).

### 2.4. SARS-CoV-2 Modulated TLR Signaling

Given that “Coronavirus disease—COVID-19” is among the most overrepresented maps and TLRs are upregulated and were reported to bind S protein, we focused on TLRs signaling using both the “Coronavirus disease—COVID-19” pathway, “Toll-like receptor signaling pathway” and canonical “NF-kappa B signaling pathway” on KEGG. We also looked at RIG-1 signaling in the “Coronavirus disease—COVID-19” pathway given the upregulation of *DDX58.* Additionally, we evaluated the transcription factors activated by MAPK in the “MAPK signaling pathway” on KEGG. The genes are shown in Table 2. We found that *TLR1*, *TLR6*, *CASP8*, *TLR4*, *MAP3K7*, *CHUK*, *RELA*, *NFKBIA*, *MAPK3*, *MAPK10*, *CXCL8*, *CCL5*, *TAB2*, *CCL2*, *CXCL10*, *MMP3*, *PTGS2*, *ATF4*, *ISG15* and *DDX58* were upregulated. On the other hand, *IKBKG*, *IKBKB*, *MAPK12*, *MAPK13*, *TRAF3*, *IKBKE*, *IRF3*, *JUN*, *FOS*, *IL1B*, *MAP2K3* and *MAP2K7* were downregulated. 

**Figure 4:**
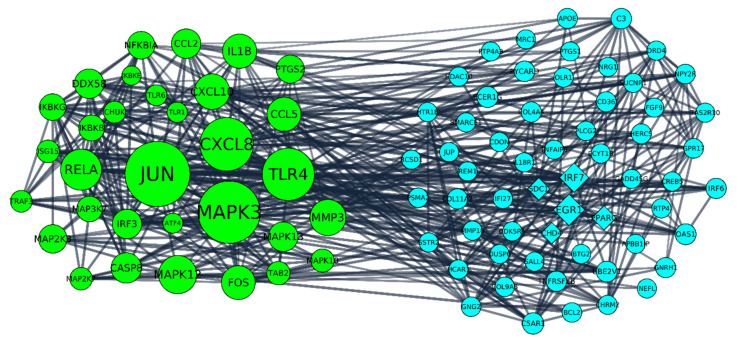
The interactomic analysis was focused on DEGs that have a fold change magnitude higher than 2 (blue DEGs on the right). Nevertheless, we also included in the analysis DEGs that are already known to be associated with immunity response selected on the bases of KEGG analysis even if only some of them have higher fold change differences (green DEGs on the left). The size of the nodes is dependent on the stress parameter so the bigger the node size, the higher the stress of that node in the network. The stress parameter gives an index of the centrality of the node in the network. Among the blue DEGs, *EGR1*, *IRF7*, *PPARG*, *SDC1* and *CHD4* in diamond shape had the five highest stress levels. For this reason, they may have a key role in immunity response to SARS-CoV-2.

Furthermore, DEGs with fold change magnitude higher than 2 were inspected. Among 860 DEGs, 396 were upregulated in HCN2-SARS-CoV-2 compared to HCN2-CTR and 464 were downregulated in HCN2-SARS-CoV-2 compared to HCN2-CTR. Thus, we performed a network analysis on STRING using DEGs with fold change magnitude higher than 2 together with DEGs selected with KEGG pathways (Table 2). We used the very strict filters high confidence score and “Experiments” and “Databases” interaction sources. *C5AR1*, *DRD4*, *COL9A3*, *MRC1*, *CREB5*, *CHRM2*, *UBE2V1*, *PYCARD*, *JUP*, *APBB1IP*, *IL18R1*, *GADD45G*, *CD36*, *PPARG*, *TAS2R10*, *TNFRSF1B*, *CDON*, *SSTR2*, *COL11A2*, *CDK5R1*, *MMP10*, *IRF6*, *PSMA2*, *RCSD1*, *BCL2*, *EGR1*, *DUSP6*, *PTGS1*, *IRF7*, *PLCG2*, *SDC1*, *APO*, *HERC5*, *OLR1*, *NPY2R*, *IFI27*, *HTR1B*, *BTG2*, *FGF9*, *SALL4*, *REM1*, *NRG1*, *SMARCE1*, *TNFAIP6*, *GPR17*, *COL4A4*, *OAS1*, *CHD4*, *RTP4*, *FCER1G*, *GNRH1*, *C3*, *NEFL*, *SUCNR1*, *HCAR1*, *GNG2*, *HDAC10*, *PCYT1B*, *PTP4A3* are DEGs with fold change magnitude higher than 2 that are the first neighbors of DEGs in Table 2. Additionally, we put a focus on the “Stress” parameter obtained after the network analysis on Cytoscape. This value gives information about the centrality of the nodes. Specifically, the stress of a node is computed counting the number of shortest paths in which the node is included. For this reason, a node with a high stress is highly involved in the biological process. In addition to the aforementioned genes, *EGR1*, *IRF7*, *PPARG*, *SDC1* and *CHD4* were the 5 DEGs with the highest stress that were not inspected in the pathways (Figure 4). 

### 2.5. SARS-CoV-2 Modulated Cytokines/Chemokines Levels in the Cell Culture Medium

**Figure 5:**
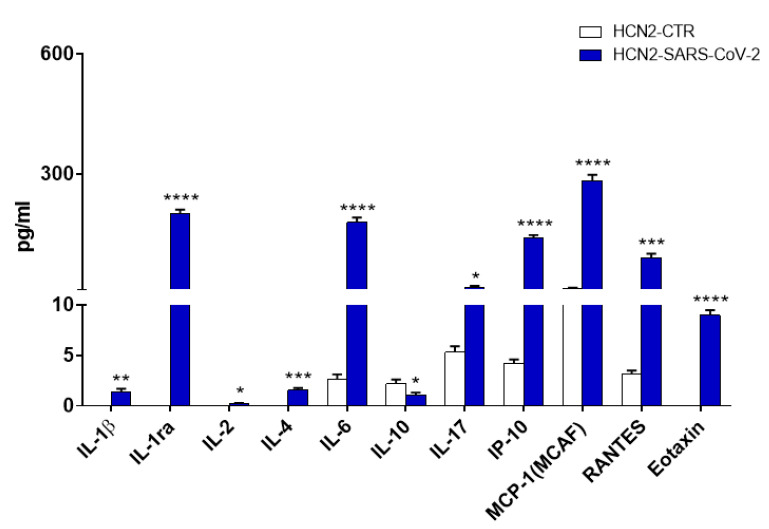
Concentration of cytokines/chemokines in cell culture supernatants 6 days post infection. * *p* < 0.05; ** *p* < 0.01; *** *p* < 0.001; **** *p* < 0.0001 compared to HCN2-CTR.

The levels of cytokines/chemokines in cell culture supernatants were evaluated in order to verify the existence of a pro-inflammatory state in HCN-2 neurons infected with SARS-CoV-2. An increase in the levels of IL-1β, IL-1ra, IL-2, IL-4, IL-6, IL-17, IP-10, MCP-1, RANTES and Eotaxin was found. On the contrary, IL-10 level decreased in HCN2-SARS-CoV-2 (Figure 5). 

IP-10 (*CXCL10*), MCP-1 (*CCL2*), and RANTES (*CCL5*) increase confirmed the results of our transcriptomic analysis, indeed the corresponding genes were upregulated (Table 3). IL-2, IL-4 and IL-10 levels showed only slight variations in their levels after infections and we did not find any significant gene expression modification for their transcripts or for their receptors. Additionally, a slight increase of IL-1β level was noted. In our transcriptomic analysis we found its downregulation, but its receptor encoded by *IL1R1* increased (Table 3). The divergent results may depend on the increase of IL-1ra, that inhibits the activity of IL-1 binding its receptor. In line with the increase in Eotaxin, IL-6 and IL-17 levels we found in the transcriptomic analysis the upregulation of eotaxin-3, the signal transducer and receptor subunit, respectively (Table 3). 

### 2.6. SARS-CoV-2 Modulated Genes Associated to COVID-19 in UniProt

The UniProt website contains the genes that are to date associated with the infection of the SARS-CoV-2 and COVID-19 outbreaks. Among them, the 28 genes represented in Table 4 were deregulated in our analysis, so they have a potential role also in HCN-2 infection. 

## 3. Discussion

A prevalence of the nervous system’s involvement in patients with COVID-19, ranging from 22.5 to 36.4% among different studies, has been described [30]. Neurological involvement of COVID-19 in neonates and children is still quite rare, but recent case reports document the potential neurologic involvement also in pediatric age [34,39]. Long period of SARS-CoV-2 infection was associated with psychiatric and cognitive disorders in adolescents and children [40]. The dormant persistence of SARS-CoV-2 in the CNS may lead to neurological complications [40]. 

In this work we aimed to study the capacity of SARS-CoV-2 to infect HCN-2 neuronal cortical cells, studying the changes in the transcriptomic profile. Indeed, it is not clear if SARS-CoV-2 can infect neuronal cells. 

### 3.1. SARS-CoV-2 Replicated in Neuronal Cells and Modulated PRR Expression

Our results showed that SARS-CoV-2 was able to replicate in HCN-2 cells as suggested by the increase in copy number of N1 and N2 genes. Our results agree with a study that showed viral particles inside a human induced pluripotent stem cells-derived brain sphere model supporting the replication of the virus [41]. On the contrary, Ramani et al. found that SARS-CoV-2 targeted neurons of 3D human brain organoids but it did not appear to efficiently replicate [42]. In parallel, Tiwari et al. found that the infection of cerebral organoids was less efficient compared to lung organoids due to low ACE2 and TMPRSS2 expression [43]. On the contrary, other studies in brain organoids indicated that SARS-CoV-2 infected choroid plexus but not neurons [44,45]. 

Interestingly, Yi et al. found ACE2 expression in brain organoids, in the somas of mature neurons, but not in neural stem cells, but SARS-CoV-2 was observed in the axons which lack ACE2 [46]. 

In our experimental model, we did not find the expression for *ACE2* and *TMPRSS2* as confirmed also by Human Protein Atlas and the Gene Expression Atlas that reported no expression in the human cerebral cortex. Additionally, Western blot analysis showed no expression of ACE2 in HCN-2 cells. ACE2 expression in neurons is not clear and may depend on the brain area. ACE2 is expressed in choroid plexus and paraventricular nuclei of the thalamus, but not in the prefrontal cortex [27] and in mature or immature olfactory receptor neurons [47]. 

KEGG “Coronavirus disease—COVID-19” pathway was among the most overrepresented together with pathways related to immune system, viral and bacterial infections, as reported also in other studies [48,49].

It is possible to speculate that also other receptors may interact with SARS-CoV-2 proteins and mediate its entry or pathogenesis. For this reason, we looked at the expression of genes belonging to known PRR families, that take part in the innate immune response. The main families of PRRs are TLRs, NLRs, RLRs, and also Scavenger receptors [50]. We found the deregulation of different scavenger receptors (Table 1) and in particular one of them, SR-B1, was shown to facilitate SARS-CoV-2 entry into ACE2-expressing cells [51]. 

A downregulation of NLRs was found in our transcriptomic analysis (Table 1). This could indicate that SARS-CoV-2 infection may induce different cell-specific pathways as suggested by Tiwari et al. that reported induction of interferons, cytokines, and chemokines and inflammasome activation in lung organoids, while in neuronal cells SARS-CoV-2 activated TLR3/7, OAS2, complement system, and apoptotic genes [43]. Instead, TLRs and RIG-1 were upregulated. In particular, TLRs seem to be involved in COVID-19 pathogenesis and emerged as potential entry factors [29,52]. 

We also found the upregulation of *DDX58*, encoding for RIG-1, a known receptor for viral RNA, that led to transcriptional induction of genes encoding type I interferons [53]. However, downstream RIG-1, the genes encoding for TRAF3, IKKε (*IKBKE*), and IRF3 were downregulated. It was already reported that SARS-CoV-2 is able to antagonize IFN-I production and signaling [54,55,56]. We also found *ISG15* upregulation, that is targeted by SARS-CoV-2 protease, to attenuate type I interferon responses [57]. Moreover, a negative regulation of RIG-I-mediated antiviral activity due to ISG15 conjugation was reported [58]. In our transcriptomic analysis interferon response seemed to be attenuated, in line with previous results in which human brain organoids were infected but no type I interferon response was detected [26]. 

### 3.2. SARS-CoV-2 Induced TLRs, NF-κB and MAPK Signaling

In our transcriptomic analysis we found the upregulation of *TLR1*, *TLR4* and *TLR6*. The expression of TLR4 was also confirmed by Western blot analysis. Interestingly, a molecular docking study indicated the binding of native spike protein of SARS-CoV-2 to TLR1, TLR4, and TLR6 and specifically, TLR4 possessed the strongest binding affinity to spike protein [59]. Other evidence suggested that SARS-CoV-2 S-protein can bind with TLR4/MD-2 complex [60] and can activate TLR4 in monocyte, neutrophil and macrophage cell lines [61]. It is important to notice that activation of TLR4 by spike protein was not regulated by ACE2 and TMPRSS2 or virus entry [61]. These results may indicate a role for TLRs in the manifestations of COVID-19 pathology as suggested by an enhancement of TLR4 mediated inflammatory signaling in PBMCs of COVID-19 patients [62]. 

The canonical pathway of TLR signaling leads to NF-κB activation, that in turn increases the expression of cytokines and chemokines. In particular, TLRs, through an activation cascade, activate TAK1 and TAB2, encoded by *MAP3K7* and *TAB2*, respectively. Activated TAK1 leads to the activation of IκB kinase (IKK), that phosphorylates and degrades the inhibitory molecule IκB [63] allowing dimerization and nuclear translocation of the p65 and p50 subunits of NF-κB. TAK1 can also activate the ERK1/2 mitogen-activated protein kinases (MAPK), JNK, and p38 [63]. Our transcriptomic analysis seemed to indicate the activation of TLR4 signaling and of its downstream mediators NF-κB and MAPK. In our transcriptomic analysis we found the upregulation of the genes encoding for TAB2 and TAK1. Moreover, we found the upregulation of *CHUK* encoding for the catalytic subunit IKKα, while *IKBKB* and *IKBKG* encoding for IKKβ and IKKγ were downregulated (Table 2). We also found the upregulation of NF-κB p65 subunit (*RELA*) and of its inhibitor IκBα (*NFKBIA*). The upregulation of *NFKBIA* is not surprising because it depends on a negative feedback loop. 

In addition, ERK (*MAPK3*) and JNK (*MAPK10*) were upregulated in our transcriptomic analysis, while p38 (*MAPK12* and *MAPK13*) and MKK3/7 (*MAP2K3* and *MAP2K7*) were downregulated (Table 2). ERK activation may prevent host cell apoptosis causing viral spread and the persistence of viral infection [64]. *MAP2K7* downregulation may depend on feedback mechanisms or on mutual regulation by the different pathways. Indeed, it is known that the NF-κB pathway is able to modulate JNK, acting on MKK7 [65]. The involvement of JNK instead of p38 may be a feature of infected neurons. A previous work reported that JNK cascade was activated in influenza A virus-infected neurons, while a delayed p38 activation was visible in astrocytes [66]. *JUN* and *FOS* were downregulated, as found also in a transcriptomic analysis of nasopharyngeal swabs of SARS-CoV-2 positive compared to negative subjects [67]. Interestingly, *JUN* transcript was restricted at post transcriptional level by SARS-CoV-2 [68], also due to its short half-life [69]. However, looking at the transcription factors activated by MAPK in KEGG, we found the upregulation of *ATF4*, reported to be activated by TLR4 pro-inflammatory signaling [70]. Interestingly, ATF4 and NF-κB were suggested as drivers of the proinflammatory cytokine response in human airway epithelial cells infected by SARS-CoV-2 [71].

These results indicated that SARS-CoV-2 infection of cortical neurons led to TLR activation, with the induction of NF-κB and MAPK signaling. The ability of SARS-CoV-2 S1 protein to induce pro-inflammatory mediators through NF-κB and JNK activation via TLR4 was also demonstrated in macrophages [72]. These results should also be confirmed in other neuronal cell lines, in order to verify if this mechanism is valid for other cortical neurons and for other neuronal cell types.

### 3.3. Cytokines and Chemokines Expression

NF-κB activation induces the expression of cytokines and chemokines. In particular, we found the upregulation of *CXCL8*, *CXCL10*, *CCL2*, *MMP3*, *CCL5*, and *PTGS2* encoding for IL-8, IP-10, MCP-1, MMP3, RANTES and COX2, respectively (Table 2). IL-8, IP-10, MCP-1 and MMP-3 serum levels were suggested as biomarkers for COVID-19 disease [73,74,75]. RANTES and COX-2 increased in SARS-CoV-2 infection [76,77]. In accordance with transcriptomic analysis, cytokines/chemokines increased in the medium (Figure 5 and Table 3). Interestingly, IL-1ra, IL-6, IL-8, MCP-1, IP-10, and Eotaxin levels were higher in children with MIS-C related to SARS-CoV-2 than in those without [78]. These results suggested that in these experimental conditions neurons produced mainly chemokines in order to recruit cells of the immune system. 

In the cell culture medium, we also found a slight increase in the levels of IL-1β. However, we found *IL1B* downregulation, but the expression of its receptor *IL1R1* was upregulated. The low levels of IL-1β may depend on the increase of IL-1ra, that binds to IL1R1 and inhibits the activity of IL-1. Additionally, an increase in IL-6 and IL-17 levels in the medium was found and in parallel their receptor subunits showed an increased expression.

We found the upregulation of the gene encoding for caspase 8, that mediates cell death and inflammation caused by SARS-CoV-2 infection of lung epithelial cells [79].

### 3.4. Network Analysis and UniProt Repository Inspection

Then, we performed a network analysis to find the genes with fold change >2 of our transcriptomic analysis that interact directly with the selected genes. In particular, we evidenced that among the interacting genes, *EGR1*, *IRF7*, *PPARG*, *SDC1* and *CHD4* were the five with a higher stress value, that indicate their relevance in connecting the regulatory molecules. These genes were all downregulated and in particular *EGR1* and *IRF7* are involved in interferon signaling, *PPARG* and *SDC1* may be involved in SARS-CoV-2 pathogenesis [80,81], while *CHD4* belonged to the pathway viral carcinogenesis reported to be significant in our transcriptomic analysis.

Moreover, in order to deepen the knowledge about the SAR-CoV-2 infection of neurons, we searched for genes known to be involved in COVID-19 reviewed by UniProt. In total, 28 genes showed a significantly changed expression and some of them were involved in interferon, cytokine, and RIG-1 signaling. We also found the upregulation of *IFNAR1*, a receptor for Type I interferon with low affinity. This may indicate also the lack of interferon, indeed, it was reported that interferon stimulation led to the downregulation of its receptor [82]. Other genes were reported to play a role in virus entry, such as *FURIN*, needed for proteolytic activation of SARS-CoV-2 [83], *BSG* encoding for CD147, with contrasting results about its role as an entry factor [84,85], and *PIKFYVE* [86]. This result indicated that also in neurons, even in the absence of ACE2, SARS-CoV-2 is able to induce transcriptional changes of genes associated to its pathogenesis.

However, it is important to notice that no cut-off was chosen to define DEGs but only genes which *q*-value was lower than 0.05 were inspected. For this reason, some of the genes present a low, even if significant, fold change. Indeed, even if a gene shows a low fold change, it can play an important role in a biological signaling.

## 4. Materials and Methods

### 4.1. Virus

SARS-CoV-2 (Human 2019-nCoV strain 2019-nCoV/Italy-INMI1, Rome, Italy) was provided by the European Virus Archive goes Global (EVAg) through the National Institute for Infectious Diseases “Lazzaro Spallanzani” IRCCS organization. The virus was expanded on Calu-3 cells (ATCC^®^ HTB-55™) and viral titers were determined by TCID_50_ endpoint dilution assay. Briefly: serial 10-fold dilutions, from 10^6^ to 10^−4^ TCDI_50_/mL (50 μL), were plated onto 96-well plates, incubated at 37 °C in 5% CO_2_ and checked daily to monitor the virus-induced cytopathic effect. Seventy-two hours post infection (hpi) viral titer was determined as previously described [87]. All the experiments with SARS-CoV-2 virus were performed in a BSL3 facility.

### 4.2. In Vitro HCN-2 SARS-CoV-2 Infection Assay

HCN-2 cells (ATCC CRL-10742) were cultured in DMEM (Euroclone, Milan, Italy) with 10% FBS medium and 100 U/mL penicillin and 100 μg/mL streptomycin, in a 25 cm^2^ culture flask. DMEM containing 100 U/mL penicillin and 100 µg/mL streptomycin was used as inoculum in the mock-infected cells. Cell cultures were infected with 1 multiplicity of infection (MOI) and incubated at 37 °C and 5% CO_2_. After 3 h cells were washed two times with lukewarm PBS and refilled with the proper growth medium (10% FBS). Optical microscope observation (ZOE™ Fluorescent Cell Imager, Bio-Rad, Hercules, CA, USA) was performed daily to investigate the cytopathic effect. SARS-CoV-2 RNA was extracted from 1, 3 and 6 days post infection (dpi) supernatant using the Maxwell^®^ RSC Instrument with Maxwell^®^ RSC Viral Total Nucleic Acid Purification Kit (Promega, Fitchburg, WI, USA). Viral RNA was reverse transcribed in a single-step RT-qPCR (GoTaq 1-Step RT-qPCR; Promega, Fitchburg, WI, USA) on a CFX96 instrument (Bio-Rad, Hercules, CA, USA) using primers specifically designed to target two regions of the nucleocapsid (N1 and N2) gene of SARS-CoV-2 (2019-nCoV CDC qPCR Probe Assay emergency kit; IDT, Coralville, IA, USA), together with primers for the human RNase P gene. Viral copy quantification was assessed by creating a standard curve from the quantified 2019-nCoV_N positive Plasmid Control (IDT, Coralville, IA, USA). The infected cells were harvested for mRNA collection 6 dpi. RNA was extracted from mock and infected HCN-2 with the acid guanidium thiocyanate–phenol-chloroform method by using the RNAzolTM B (Tel-Test, Inc., Friendswood, TX, USA) and dissolved in RNAse-free water.

### 4.3. RNA-seq Analysis

The library preparation was performed following the TruSeq RNA Exome protocol (Illumina, San Diego, CA, USA) following the instruction, as previously described by Silvestro et al. [88]. The Illumina MiSeq Instrument was used to obtain the raw data from the libraries. The tool fastQC (version 0.11.5, Babraham Institute, Cambridge, UK) was used to check the quality parameters of the raw data in Fastq format. We counted 17,605,130 reads for the HCN2-CTR and 10,128,701 reads for HCN2-SARS-CoV-2. The low quality bases and the adapters were filtered out by Trimmomatic (version 0.38, Usadel Lab, Aachen, Germany) [89]. The remaining reads were aligned against the GRCh38 version of the human reference genome with Spliced Transcripts Alignment to a Reference (STAR) RNA-seq aligner (version 2.7.3a, New York, NY, USA) [90]. We checked for coverage of bam files using the tool samtools [91] with the command depth. We observed a mean coverage of 4.02624 for HCN2-CTR and of 2.71703 for HCN2-SARS-CoV-2. Then, the python package htseq-count (version 0.6.1p1, European Molecular Biology Laboratory (EMBL), Heidelberg, Germany) [92] associated the counts to each transcript and the package DESeq2 of Bioconductor [93] were used to compute the analysis of differentially expressed genes in R (version 3.6.3, R Core Team). No fold change threshold was used to discard the DEGs but the post hoc Benjamini–Hochberg procedure was adopted to adjust the *p*-value and discard DEGs with a *q*-value higher than 0.05.

### 4.4. In Silico Data Analysis

The Human Protein Atlas (http://www.proteinatlas.org (accessed on 11 June 2021)) [94] and the Gene Expression Atlas (https://www.ebi.ac.uk/gxa/home (accessed on 11 June 2021)) [95] databases were used to confirm the absence of ACE2 and TMPRSS2 in the human cerebral cortex. In R, we searched for the pathways overrepresented in the Kyoto Encyclopedia of Genes and Genomes (KEGG) database [96] using the enirchKEGG function of the library clusterProfiler [97]. The name of each DEG was converted in its related entrez using the ensembl package biomaRt [98]. The Benjamini–Hochberg method was adopted to reduce the false positives. Only the maps with a *q*-value lower than 0.05 were represented in the Bubble Plot realized with the library ggplot2 [99]. To have a clear representation of the maps we replaced the *q*-value with a score −log(*q*-value). Additionally, KEGG was used to inspect the curated biological pathways related to Coronavirus Disease—COVID-19 (hsa05171), Toll-like receptor signaling pathway (hsa04620), NF-kappa B signaling pathway (hsa04064). Then, a final interactomic analysis was carried out with STRING database [100]. The high confidence (0.700) level and “Experiments” and “Databases” interaction sources were required. Finally, CYTOSCAPE (version 3.8.2, Institute for Systems Biology, Seattle, WA, USA) [101] was used to customize the network and perform network analysis. The HUGO Gene Nomenclature Committee (HGNC) website [102] was used to characterize the PRRs in the Toll like receptors (group 948), NLR family (group 666) and Scavenger receptors (1253). Additionally, the category Rig-like receptors from the PRRDB 2.0: Pattern Recognition Receptor Database [103] was included. The COVID-19 section of the UniProt (https://covid-19.uniprot.org/ (accessed on 11 June 2021)) [104] website contains the reviewed proteins associated with SARS-CoV-2.

### 4.5. Protein Extraction and Western Blot Analysis

Human Lung Carcinoma Cells (A549) Expressing Human Angiotensin-Converting Enzyme 2 (hACE2) (Catalog No. NR-53821) were purchased by Bei Resources. A549-hACE2 were grown in Dulbecco’s Modified Eagle’s Medium (DMEM) (Euroclone, Milan, Italy) containing 4 mM L-glutamine, supplemented with 10% FBS, 100 U/mL penicillin and 100 μg/mL streptomycin and cultured at 37 °C and 5% CO_2_. HCN-2 cells, A549 cells and A549-hACE2 were harvested and proteins were extracted using RIPA following manufacturer protocol. Protein concentrations were evaluated using the Bradford assay (Bio-Rad, Hercules, CA, USA). Proteins were heated for 5 min at 95 °C, subjected to SDS-polyacrylamide gel electrophoresis (SDS-PAGE) and transferred onto a PVDF membrane (Immobilon–P, Millipore, Burlington, MA, USA). Membranes were blocked in 5% skim milk in PBS for 1 h at room temperature followed by incubation overnight at 4 °C with the following primary antibodies: ACE-2 (1:1000; Abcam, Cambridge, UK), TLR4 (1:500; Abcam, Cambridge, UK). Then, membranes were washed in PBS 1 × and incubated with HRP-conjugated anti-rabbit or anti-mouse IgG secondary antibody (1:2000; Santa Cruz Biotechnology Inc, Dallas, TX, USA) for 1 h at room temperature. The relative expression of protein bands was visualized using an enhanced chemiluminescence system (Luminata Western HRP Substrates, Millipore, Burlington, MA, USA) and protein bands were acquired with ChemiDoc™ MP System (Bio-Rad).

### 4.6. Cytokine and Chemokine Measurement by Multiplex Assay

Concentration of cytokines/chemokines was assessed in cell culture supernatants 6 days post infection by using immunoassays formatted on magnetic beads (Bio-Plex Pro Human Cytokine 27-plex Assay #M500KCAF0Y) (Bio-Rad, Hercules, CA, USA), according to manufacturer’s protocol via Luminex 100 technology (Luminex, Austin, TX, USA). Briefly, the capture antibody-coupled beads are first incubated with antigen standards or samples for a specific time. The plate is then washed to remove unbound materials, followed by incubation with biotinylated detection antibodies. After washing away the unbound biotinylated antibodies, the beads are incubated with a reporter streptavidin-phycoerythrin conjugate (SA-PE). Following removal of excess SA-PE, the beads are passed through the array reader, which measures the fluorescence of the bound SA-PE. For the targets over-range an arbitrary value of 4000 pg/mL is assigned, while 0 pg/mL is attributed to values below limit of detection.

### 4.7. Statistical Analysis

GraphPad Prism 6.0 software (GraphPad Software, La Jolla, CA, USA) was used to perform the statistical analysis. The multiple comparison was performed using one-way ANOVA test and the Bonferroni post hoc test. The comparison between two groups was performed using Student’s *t*-test. A *p* value less than or equal to 0.05 was considered statistically significant. 

## 5. Conclusions

This study indicated that SARS-CoV-2 induced a pro-inflammatory response in cortical neurons even if ACE2 receptor is not expressed. The transcriptomic analysis evidenced that different PPRs are deregulated and in particular TLR1, TLR4 and TLR6 signaling is upregulated leading to the activation of NF-κB and MAPK signaling. As a consequence, COX-2, MMP3 and chemokines, that may represent a damage signal in order to enroll immune system cells, were upregulated. Moreover, we observed a deregulation of type I interferon pathway, supporting the idea that SARS-CoV-2 attenuates this pathway to evade the immune system. These results evidenced that SARS-CoV-2 can activate a pro-inflammatory state also in cells not expressing ACE2. 

## Figures and Tables

**Table 1:** DEGs identified as pattern recognition receptors.

Gene	HCN2-CTR Mean Counts	HCN2-SARS-CoV-2 Mean Counts	Log Fold Change	*q*-Value	PRRs Type
*AGER*	22.40	6.250	−1.84	6.61 × 10^−04^	Scavenger receptors
*CD163L1*	590.42	754.98	0.35	1.72 × 10^−09^	Scavenger receptors
*CD36*	116.80	7.50	−3.96	1.63 × 10^−19^	Scavenger receptors
*DDX58*	393.61	507.49	0.37	4.69 × 10^−07^	Rig-like receptors
*MEGF10*	36.80	58.75	0.67	3.86 × 10^−03^	Scavenger receptors
*MRC1*	1.60	17.50	3.45	3.24 × 10^−05^	Scavenger receptors
*NLRC5*	622.42	218.74	−1.51	2.73 × 10^−68^	NLR family
*NLRP1*	228.01	116.25	−0.97	1.62 × 10^−14^	NLR family
*OLR1*	2.40	10.00	2.06	7.22 × 10^−03^	Scavenger receptors
*SCARB1*	198.41	267.49	0.43	2.55 × 10^−05^	Scavenger receptors
*SCARB2*	1432.04	1694.95	0.24	2.80 × 10^−10^	Scavenger receptors
*SCARF1*	11.20	1.25	−3.16	6.91 × 10^−03^	Scavenger receptors
*SSC5D*	1382.44	1993.69	0.53	8.79 × 10^−49^	Scavenger receptors
*TLR1*	11.20	32.50	1.54	2.38 × 10^−05^	Toll-like receptors
*TLR4*	514.41	629.98	0.29	6.88 × 10^−06^	Toll-like receptors
*TLR6*	46.40	106.25	1.2	5.09 × 10^−11^	Toll-like receptors

Log fold change differences for DEGs identified as pattern recognition receptors between HCN2-SARS-CoV-2 and HCN2-CTR. For each DEG, we included the mean counts computed after DESeq2 normalization in both conditions. The PRR classes to which they belong are highlighted.

**Table 2:** DEGs included in the immune response to SARS-CoV-2 in HCN-2 cells.

Gene	HCN2-CTR Mean Counts	HCN2-SARS-CoV-2 Mean Counts	Log Fold Change	*q*-Value
*TLR1*	11.20	32.50	1.54	2.38 × 10^−05^
*TLR6*	46.40	106.25	1.20	5.09 × 10^−11^
*CASP8*	148.00	184.99	0.32	1.00 × 10^−02^
*TLR4*	514.41	629.98	0.29	6.88 × 10^−06^
*MAP3K7*	359.21	416.24	0.21	9.48 × 10^−03^
*IKBKG*	52.00	36.25	−0.52	4.35 × 10^−02^
*CHUK*	618.42	752.48	0.28	1.73 × 10^−06^
*IKBKB*	740.82	544.98	−0.44	7.54 × 10^−13^
*RELA*	465.61	554.98	0.25	2.89 × 10^−04^
*NFKBIA*	724.82	986.22	0.44	4.94 × 10^−18^
*MAPK3*	1052.83	1157.47	0.14	4.73 × 10^−03^
*MAPK12*	76.80	32.50	−1.24	2.20 × 10^−07^
*MAPK13*	62.40	42.50	−0.55	1.80 × 10^−02^
*MAPK10*	155.20	197.49	0.35	3.97 × 10^−03^
*CXCL8*	25.60	73.75	1.53	7.78 × 10^−11^
*CCL5*	0.00	16.25	6.79	1.72 × 10^−05^
*TAB2*	920.83	1143.72	0.31	3.83 × 10^−11^
*TRAF3*	466.41	366.24	−0.35	8.36 × 10^−06^
*IKBKE*	101.60	76.25	−0.41	1.97 × 10^−02^
*IRF3*	239.21	147.50	−0.70	2.30 × 10^−09^
*CCL2*	945.63	4157.38	2.14	0
*CXCL10*	0.80	5.00	2.64	4.04 × 10^−02^
*MMP3*	1.60	232.49	7.18	6.30 × 10^−22^
*PTGS2*	21.60	343.74	3.99	3.73 × 10^−82^
*JUN*	413.61	209.99	−0.98	6.81 × 10^−26^
*FOS*	172.00	11.25	−3.93	1.75 × 10^−28^
*IL1B*	47.20	8.75	−2.43	1.65 × 10^−08^
*ATF4*	1512.04	1806.2	0.26	5.75 × 10^−12^
*DDX58*	393.61	507.49	0.37	4.69 × 10^−07^
*MAP2K3*	1580.84	1204.97	−0.39	2.23 × 10^−21^
*MAP2K7*	122.40	93.75	−0.38	1.66 × 10^−02^
*ISG15*	52.80	87.50	0.73	1.20 × 10^−04^

Log fold change differences for DEGs involved in immunity obtained between HCN2-SARS-CoV-2 and HCN2-CTR. For each DEG, we included the mean counts computed after DESeq2 normalization in both conditions.

**Table 3:** DEGs encoding for cytokines/chemokines and their receptors.

Gene	Protein	HCN2-CTR Mean Counts	HCN2-SARS-CoV-2 Mean Counts	Log Fold Change	*q*-Value
*IL1B*	Interleukin-1 β	47.20	8.75	−2.43	1.65 × 10^−08^
*IL1R1*	Interleukin-1 receptor type 1	159.20	456.24	1.52	1.89 × 10^−62^
*IL6ST*	Interleukin-6 receptor subunit beta	1941.66	2153.69	0.15	1.47 × 10^−05^
*IL17RA*	Interleukin-17 receptor A	340.81	414.99	0.28	4.85 × 10^−04^
*CCL5*	C-C motif chemokine 5 (RANTES)	0.00	16.25	6.79	1.72 × 10^−05^
*CCL2*	C-C motif chemokine 2 (MCP-1)	945.63	4157.38	2.14	0
*CXCL10*	C-X-C motif chemokine 10 (IP-10)	0.80	5.00	2.64	4.04 × 10^−02^
*CCL26*	C-C motif chemokine 26 (Eotaxin-3)	8.80	46.25	2.39	6.93 × 10^−11^

Log fold change differences for DEGs encoding for cytokines/chemokines and their receptors between HCN2-SARS-CoV-2 and HCN2-CTR. For each DEG, we included the mean counts computed after DESeq2 normalization in both conditions.

**Table 4:** DEGs associated with SARS-CoV-2 infection in UniProt.

Gene	HCN2-CTR Mean Counts	HCN2-SARS-CoV-2 Mean Counts	Log Fold Change	*q*-Value
*APOE*	80.00	2.50	−5.00	5.65 × 10^−11^
*BSG*	1677.65	2341.18	0.48	3.23 × 10^−48^
*CTSL*	911.23	679.98	−0.42	2.13 × 10^−14^
*DDX1*	1523.24	1806.20	0.25	4.24 × 10^−11^
*DHX58*	46.40	21.25	−1.13	2.26 × 10^−04^
*EEF1A1*	29,637.64	24,050.57	−0.30	4.10 × 10^−240^
*FURIN*	693.62	1074.97	0.63	1.55 × 10^−36^
*HIF1A*	13,913.99	17,284.51	0.31	1.18 × 10^−157^
*HLA-A*	3641.70	4224.88	0.21	3.09 × 10^−19^
*HLA-E*	2016.86	1858.70	−0.12	1.27 × 10^−03^
*IFITM2*	540.02	608.73	0.17	1.04 × 10^−02^
*IFNAR1*	234.41	281.24	0.26	8.82 × 10^−03^
*IKBKG*	52.00	36.25	−0.52	4.35 × 10^−02^
*IL17RA*	340.81	414.99	0.28	4.85 × 10^−04^
*IL6ST*	1941.66	2153.69	0.15	1.47 × 10^−05^
*IRF3*	239.21	147.50	−0.70	2.30 × 10^−09^
*IRF7*	11.20	2.50	−2.16	1.17 × 10^−02^
*ISG15*	52.80	87.50	0.73	1.20 × 10^−04^
*NLRP1*	228.01	116.25	−0.97	1.62 × 10^−14^
*NUP98*	1358.44	1553.71	0.19	1.88 × 10^−06^
*PHB*	1110.43	1261.21	0.18	5.49 × 10^−05^
*PHB2*	1071.23	861.23	−0.31	3.61 × 10^−10^
*PIKFYVE*	884.83	1006.22	0.19	3.07 × 10^−04^
*PPIA*	29,713.64	29,005.43	−0.03	1.71 × 10^−04^
*SMAD3*	1147.23	459.99	−1.32	1.49 × 10^−106^
*SMPD1*	838.42	956.22	0.19	3.25 × 10^−04^
*VAMP8*	51.20	75.00	0.55	6.78 × 10^−03^
*VPS41*	872.82	1321.21	0.60	1.32 × 10^−40^

Log fold change differences between HCN2-SARS-CoV-2 and HCN2-CTR for DEGs associated with SARS-CoV-2 infection in UniProt. For each DEG, we included the mean counts computed after DESeq2 normalization in both conditions.

## Data Availability

The data presented in this study are openly available in the NCBI Sequence Read Archive at BioProject accession number PRJNA742373.
